# Purpura Fulminans in a 22-Month-Old Child: Early Recognition and Favorable Outcome Without Limb Loss

**DOI:** 10.7759/cureus.110715

**Published:** 2026-06-12

**Authors:** Hoor Hamidoglu, Roa'a Abu Tawileh, Sana Wasim, Raihanah Alshaibah, Mariam Musabeh Mohamed Alshehhi

**Affiliations:** 1 Medicine, University of Sharjah, Sharjah, ARE; 2 Pediatrics Emergency, Al Qassimi Women's and Children's Hospital, Sharjah, ARE; 3 Pediatrics Emergency, Al Qassimi Women's and Children's Hospital, Emirates Health Services, Sharjah, ARE

**Keywords:** disseminated intravascular coagulation, meningococcemia, pediatric sepsis, protein c deficiency, purpura fulminans

## Abstract

Purpura fulminans (PF) is a rare but life-threatening complication of meningococcal sepsis marked by rapidly progressive hemorrhagic skin lesions, disseminated intravascular coagulation, and microvascular thrombosis, often leading to high mortality and limb loss. We report a previously healthy 22-month-old boy who presented with fever, followed by rapidly evolving ecchymotic lesions and laboratory evidence of septic shock with disseminated intravascular coagulopathy (DIC) and markedly reduced protein C levels; blood culture and cerebrospinal fluid (CSF) polymerase chain reaction (PCR) confirmed *Neisseria meningitidis*. He was managed promptly with intravenous antibiotics, fluid resuscitation, vasopressors, and blood products. Therapeutic heparin was initiated as an individualized adjunct during the compensated phase of shock, although its benefit in this context remains uncertain. Early recognition and multidisciplinary intervention were associated with rapid stabilization and complete recovery without necrosis, limb loss, neurological deficits, or growth abnormalities. This case underscores the critical importance of early dermatologic recognition and aggressive management to prevent irreversible complications.

## Introduction

Meningococcemia is a rapidly progressive bloodstream infection caused by *Neisseria meningitidis* and represents a severe form of invasive meningococcal disease (IMD). The global incidence of IMD varies geographically, ranging from approximately less than one to 10.2 cases per 100,000 population annually, with the highest burden in children under five years of age [1].

IMD typically begins abruptly with systemic symptoms, followed by the rapid appearance of petechiae and purpuric rash. Although it classically presents as meningitis, meningococcemia may occur without overt meningeal signs, particularly in children, and may initially present with nonspecific signs of sepsis [2]. Clinical deterioration may occur rapidly, resulting in septic shock, multiorgan failure, disseminated intravascular coagulopathy (DIC), which involves widespread clotting activation leading to organ damage, and purpura fulminans (PF), even with prompt antibiotic therapy [3].

PF is a rare but life-threatening thrombotic complication of invasive infection in children, with an estimated incidence of around 0.3-0.5 per 100,000 children annually. It is seen mainly in infants and young children, most often in association with severe bacterial sepsis, particularly meningococcal disease. Despite advances in intensive care, reported mortality remains substantial at approximately 15-25%. Among survivors, long-term morbidity is frequent, and a proportion (about 5-20%) require surgical intervention, including skin grafting or limb amputation, due to extensive tissue necrosis [4].

The pathophysiology involves endotoxin-mediated cytokine activation leading to endothelial injury and profound activation of the coagulation cascade. A central mechanism is consumption and dysfunction of natural anticoagulant pathways - especially protein C- resulting in uncontrolled thrombin generation, dermal vessel thrombosis, tissue ischemia, and necrosis [5]. In children, meningococcemia complicated by PF is associated with high mortality and substantial long-term morbidity, including limb loss and growth-related orthopedic sequelae [6].

Here, we present a case of a 22-month-old child who developed meningococcal PF. This case is notable for severe coagulopathy with a relatively preserved platelet count, early recognition during the compensated stage of shock, and complete limb salvage without tissue loss. Given that pediatric PF carries high morbidity and a significant risk of tissue necrosis and amputation, this case highlights a potential early therapeutic window and the possible role of individualized adjunctive therapies.

## Case presentation

A previously healthy 22-month-old male presented to the emergency department with a three-day history of rhinorrhea, followed by one day of fever, poor oral intake, decreased activity, irritability, reduced sleep, and two episodes of watery diarrhea. He had received paracetamol and ibuprofen at home without improvement. During emergency department evaluation, approximately one hour after the initial encounter, he developed new-onset petechiae and bruising that began on the face and rapidly progressed to the lower limbs (Figures 1-3). There was no history of trauma, seizures, loss of consciousness, vomiting, abdominal pain, recent travel, or prior similar episodes. He is fully vaccinated based on the UAE immunization schedule. His past medical and surgical histories were unremarkable.

**Figure 1:**
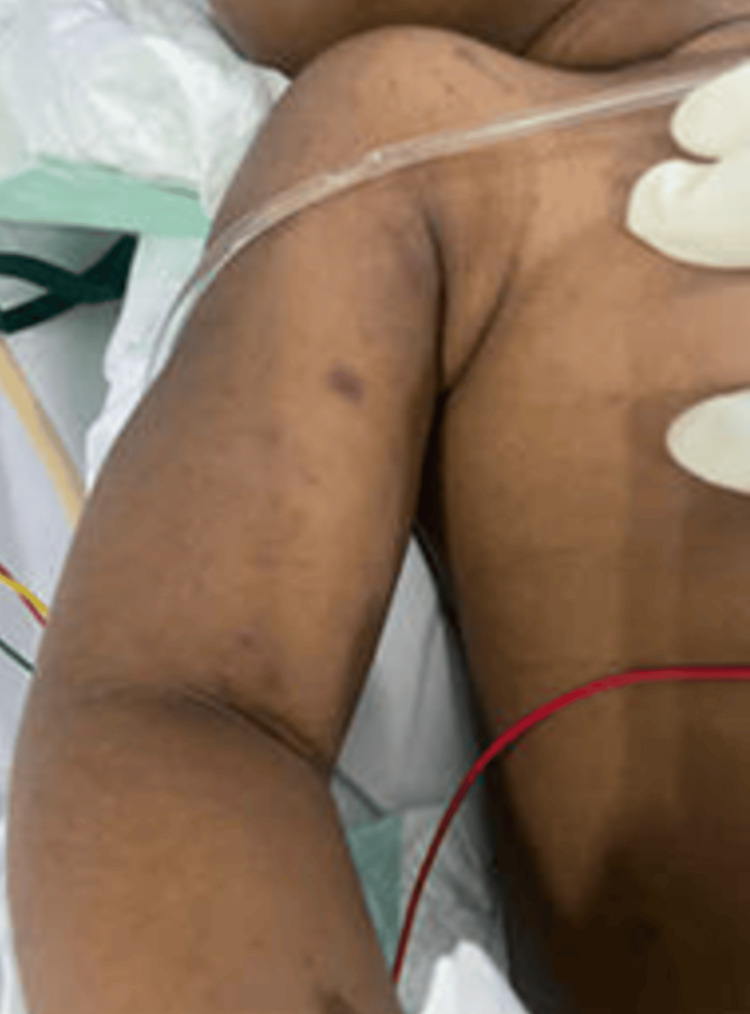
Purpuric and ecchymotic lesions over the upper limb

**Figure 2:**
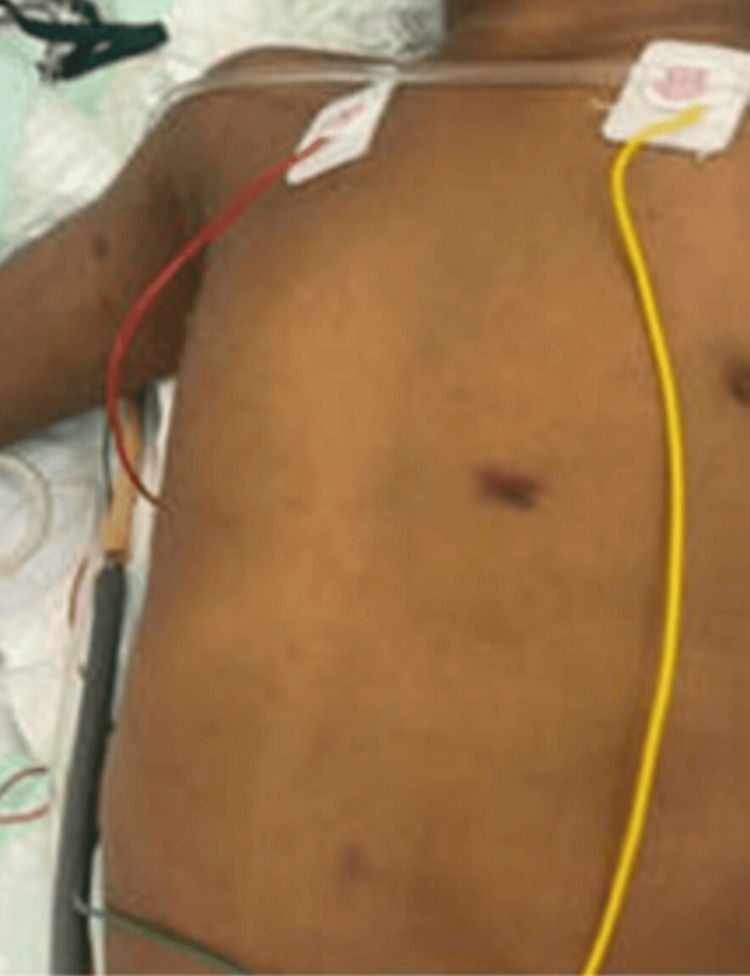
Discrete ecchymotic patches over the anterior chest wall

**Figure 3:**
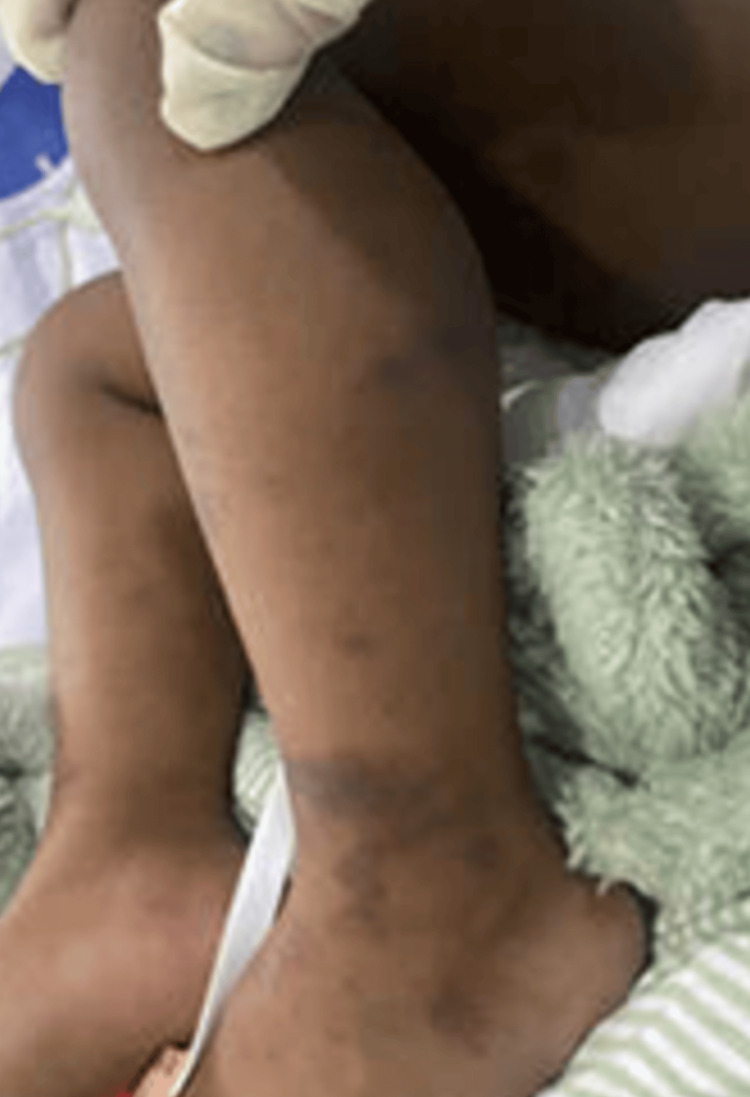
Purpuric lesions over the lower extremity, no evidence of skin necrosis

On reassessment, he appeared tired, lethargic, and irritable. Vital signs demonstrated age-appropriate blood pressure (94/63 mmHg), with marked tachycardia (180 beats/min) and tachypnea (46 breaths/min) for age, while oxygen saturation remained normal at 100% on room air. He was afebrile at that time. Capillary refill time and peripheral pulses were normal. Skin examination revealed evolving bruising and ecchymotic non-blanching lesions over the face and left lower limb. Chest and abdominal examinations were unremarkable. No focal neurological deficits or meningeal signs were present.

Laboratory investigations showed leukopenia (WBC: 4 × 10^9^/L), hemoglobin of 12.7 g/dL, and thrombocytopenia (111 × 10^9^/L), with elevated inflammatory markers (CRP: 87 mg/L, procalcitonin: > 75 ng/mL) and metabolic acidosis (venous pH: 7.2, lactate: 7 mmol/L). Coagulation studies were consistent with disseminated intravascular coagulation (DIC) (prothrombin time (PT): 19.6 s, activated partial thromboplastin time (aPTT): 44.7 s, INR: 1.73, D-dimer: > 35.2 mg/L, fibrinogen: 1.34 g/L), and natural anticoagulants were reduced (protein C: 15%, protein S: 30%). Renal function was impaired (creatinine: 101.03 µmol/L, BUN: 11.40 mmol/L), and liver enzymes were elevated. Thrombophilia and autoimmune screening, including factor V Leiden and anticardiolipin antibodies, were performed to exclude underlying hypercoagulable predispositions; all results were negative. Rapid influenza testing was negative as well (Table 1).

**Table 1:** Initial lab investigations WBC, white blood cell count; CRP, C-reactive protein; PT, prothrombin time; aPTT, activated partial thromboplastin time; INR, international normalized ratio; BUN, blood urea nitrogen. D-dimer was markedly elevated (>35.2 mg/L), representing more than a 70-fold increase above the upper limit of normal (<0.5 mg/L), consistent with severe activation of coagulation and fibrinolysis. Protein C level was profoundly reduced (15%), a level associated with increased risk of severe thrombotic complications and purpura fulminans. Protein S was also markedly decreased (30%), further supporting acquired consumption of natural anticoagulants in disseminated intravascular coagulation.

Parameter	Lab Value	Normal Range
Hematology		
WBC	4 × 10⁹/L	4.5-11.0 × 10⁹/L
Hemoglobin	12.7 g/dL	10.5-13.5 g/dL
Platelets	111 × 10⁹/L	150-400 × 10⁹/L
Inflammatory Markers		
CRP	87 mg/L	< 5 mg/L
Procalcitonin	> 75 ng/mL	< 0.1 ng/mL
Blood Gas/Metabolic		
Venous pH	7.2	7.35-7.45
Lactate	7 mmol/L	0.5-2.2 mmol/L
Coagulation Profile		
PT	19.6 s	9-14 s
aPTT	44.7 s	25-35 s
INR	1.73	0.8-1.2
D-dimer	> 35.2 mg/L	< 0.5 mg/L
Fibrinogen	1.34 g/L	2.0-4.0 g/L
Natural Anticoagulants		
Protein C	15%	70-140%
Protein S	30%	60-140%
Renal Function Tests		
Creatinine	101.03 µmol/L	44-106 µmol/L
BUN	11.40 mmol/L	2.5-7.1 mmol/L
Others		
Factor V Leiden	Negative	Negative
Anticardiolipin Ab	Negative	Negative
Rapid influenza	Negative	Negative

In the emergency department, he received intravenous fluids (10 mL/ kg, 100 mL over one hour) and was started empirically on intravenous ceftriaxone (100 mg/kg) and vancomycin (20 mg/kg). Blood cultures were obtained. He was admitted to the pediatric intensive care unit (PICU) with a working diagnosis of compensated septic shock complicated by DIC and PF. Immediately upon transfer to the PICU, norepinephrine infusion was initiated at 0.025 mcg/kg/min for one day and continued until the morning of the second hospital day. He received transfusions of fresh frozen plasma (10-15 mL/kg ), platelets, and packed red blood cells. In addition, one dose of albumin 11 g over four hours with Lasix 10 mg IV mid-transfusion was administered to address marked hypoalbuminemia (18 g/L), contributing to intravascular oncotic support in the setting of capillary leak associated with septic shock. Following stabilization and transfer to the pediatric ward after five days. Respiratory support with high-flow nasal cannula (10 L, FiO₂ 40%) was provided and weaned to room air the same day.

Heparin infusion was initiated as part of the bundle care of central venous line patency to reduce the risk of catheter-associated thrombosis. Following hematology consultation, continuation of heparin was recommended despite it not being part of the standard protocol, due to concern for microthrombosis formation. The infusion was commenced at 10 IU/ kg/hour with dose titration guided by anti-factor Xa levels. The rate was gradually increased to 17 IU/ kg/ hour to achieve and maintain therapeutic anticoagulation while balancing the risk of bleeding. The patient was closely monitored through therapy with serial laboratory assessments.

Blood culture results became available on the second hospital day and grew *N. meningitidis*, sensitive to ceftriaxone and meropenem. Intravenous ceftriaxone was continued for a total of five days, while IV meropenem (40 mg/kg) was administered for four days. Vancomycin was discontinued after two days due to a lack of evidence for resistant Gram-positive infection. Lumbar puncture performed after two days of antibiotic therapy showed cerebrospinal fluid PCR positive for *N. meningitidis *with otherwise unremarkable CSF indices and negative culture. Brain MRI was normal (Figure 4). Venous Doppler of the lower limbs, performed due to swelling and tenderness, showed no deep vein thrombosis (Figures 5-6). The patient became clinically stable with improvement of skin lesions and limb swelling. Serial laboratory trends during hospitalization were reviewed and are presented to demonstrate the temporal evolution of inflammatory and coagulation parameters in response to treatment (Table 2).

**Figure 4:**
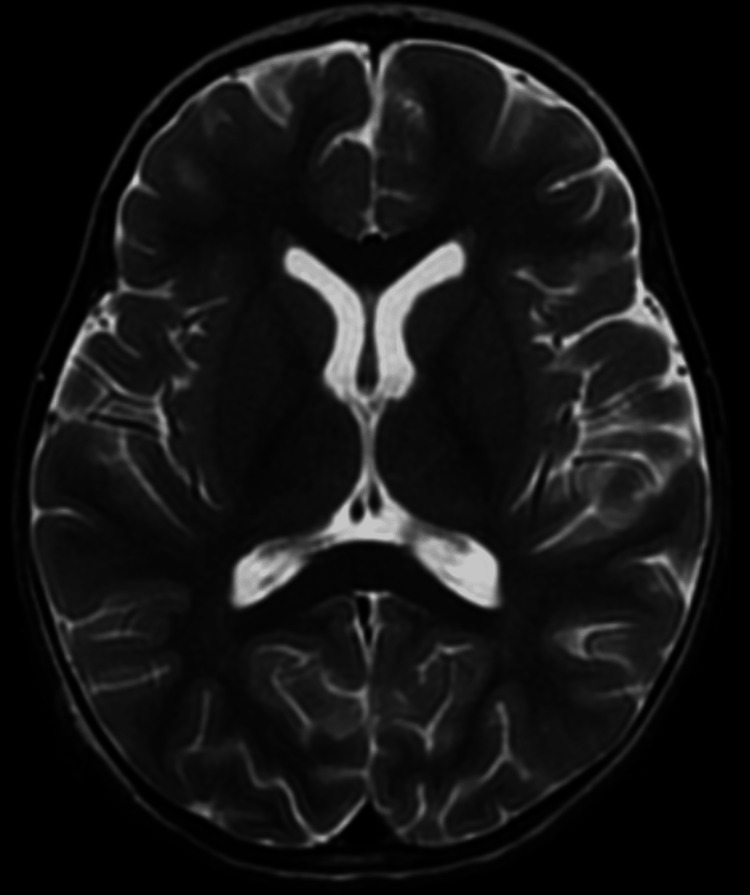
Normal brain MRI scan Magnetic resonance imaging (MRI) of the brain performed to evaluate for central nervous system complications associated with meningococcemia and purpura fulminans, including cerebral venous thrombosis, ischemic infarction, intracranial hemorrhage, or septic embolic lesions. No acute intracranial abnormalities were identified.

**Figure 5:**
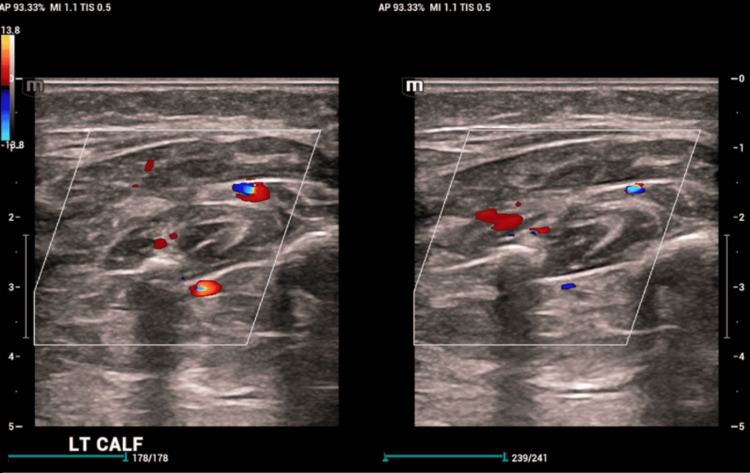
Venous Doppler without obstruction or deep venous thrombosis of the left calf veins

**Figure 6:**
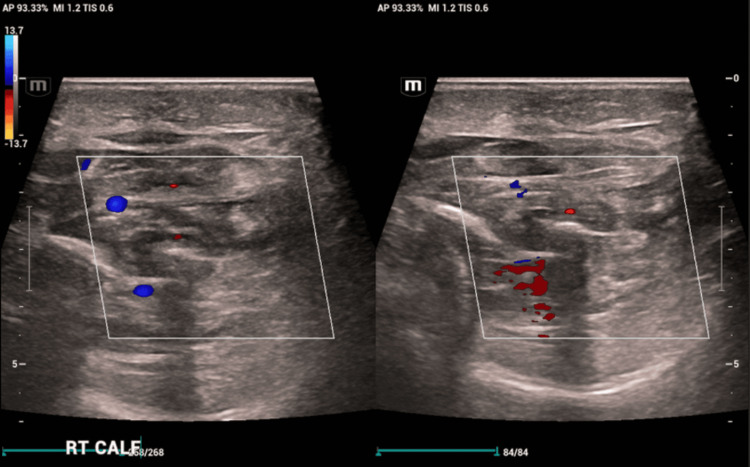
Venous Doppler without obstruction or deep venous thrombosis of the right calf veins

**Table 2:** Serial trends in hematological coagulation and perfusion markers during hospitalization Serial changes in platelet count, coagulation profile (PT, PTT, INR), lactate, and D-dimer from admission to discharge, showing progressive normalization of coagulation status and tissue perfusion with clinical recovery.

Parameter	Admission	Day 3	Day 6	Discharge
Platelets	111 × 10⁹/L	64 × 10⁹/L	97 × 10⁹/L	433 × 10⁹/L
PT	19.6 s	12.6 s	11.4 s	Normal
PTT	44.7 s	31.1 s	29.6 s	Normal
INR	1.73	1.09	0.98	Normal
Lactate	7 mmol/L	1.8 mmol/L	1.2 mmol/ L	Normal
D-dimer	> 35.2 mg/L	7.9 mg/L	5.52 mg/L	3.31 mg/L

Plastic surgery consultation was obtained during hospitalization; however, no debridement or reconstructive intervention was required. With clinical improvement, normalization of laboratory parameters, and a negative Doppler study for deep vein thrombosis, the unfractionated heparin infusion was gradually tapered and discontinued on day five. Ongoing surveillance was maintained using D-dimer levels as part of follow-up monitoring. The patient was discharged after a total hospital stay of nine days. At discharge, he was afebrile, hemodynamically stable, and moving all limbs. There was no progression to skin necrosis, ulceration, or gangrene observed, and only residual discoloration of some lesions remained.

At outpatient follow-up, conducted one week after discharge, there were no neurological sequelae, no motor deficits, and no evidence of cognitive impairment. Limb growth and perfusion remained normal. Given the short duration of follow-up, long-term orthopedic monitoring was arranged to evaluate for potential late growth plate disturbances, which have been described in survivors of meningococcal PF.

## Discussion

PF is a rare but life-threatening thrombotic complication of severe infection, most commonly associated with invasive meningococcal disease in children. The reported incidence is approximately 0.3-0.5 per 100,000 children annually, with mortality rates ranging from 12-25% despite advances in intensive care management [4]. Survivors frequently experience significant long-term morbidity, including skin scarring, growth disturbances, and limb amputation secondary to extensive tissue necrosis [6]. Acute infectious PF is classically associated with *N. meningitidis*, although congenital and post-infectious forms related to protein C or protein S deficiency have also been described [7].

The clinical presentation of our patient was consistent with the early evolution of meningococcal PF described in previous literature. Similar to reported pediatric cases, the illness began with fever and nonspecific systemic symptoms, followed by the rapid development of petechial and ecchymotic skin lesions [8]. The progression of the rash toward confluent purpura represents the characteristic dermatologic evolution of meningococcal septicemia and reflects underlying dermal vascular thrombosis and DIC [5,8]. Importantly, our patient presented during a compensated phase of shock before the development of refractory hypotension or peripheral gangrene. Early recognition of evolving ecchymotic lesions in the emergency department prompted immediate escalation of care and likely contributed to the favorable outcome observed.

The laboratory findings in this case further supported the diagnosis of meningococcal PF with consumptive coagulopathy. Previous studies have identified severe thrombocytopenia, particularly platelet counts below 50 × 10⁹/L, as a major predictor of mortality and permanent deformity in septic PF [9]. Additional poor prognostic markers include elevated D-dimer levels, reduced protein C activity, and development of symmetrical peripheral gangrene [10]. Our patient demonstrated laboratory evidence of DIC and markedly reduced protein C levels; however, platelet counts remained relatively preserved at 111 × 10⁹/L. Compared with more severe reported cases, this relative preservation of platelet count may have limited the extent of microvascular thrombosis and reduced progression to irreversible tissue necrosis.

The diagnostic approach in our case was also notable. Blood cultures obtained early during admission confirmed *N. meningitidis*, while cerebrospinal fluid (CSF) polymerase chain reaction (PCR) remained positive despite sterile CSF cultures after antibiotic initiation. This finding is consistent with previous studies demonstrating that CSF PCR maintains high sensitivity even after early antimicrobial administration, whereas culture sensitivity declines substantially following antibiotic exposure [11]. In critically ill patients who receive empiric antibiotics before lumbar puncture or culture collection, PCR therefore plays an essential role in confirming meningococcal disease.

Management of meningococcal PF required rapid multidisciplinary intervention focused on antimicrobial therapy, hemodynamic stabilization, and correction of coagulopathy. Consistent with current recommendations, our patient received immediate broad-spectrum intravenous antibiotics, aggressive fluid resuscitation, vasopressor support, and transfusion therapy, including fresh frozen plasma and cryoprecipitate [4]. Early antimicrobial administration is particularly important in meningococcal disease, as treatment delays have been associated with increased mortality [4].

Adjunctive therapies in PF remain controversial. Protein C depletion is recognized as a major contributor to the dysregulated coagulation and microvascular thrombosis observed in meningococcal PF [5,7]. Although several pediatric studies have reported improvement in coagulation parameters following protein C replacement therapy, randomized data demonstrating mortality benefit remain limited [12-14]. Therapeutic anticoagulation with heparin is similarly individualized and lacks standardized recommendations; however, it may theoretically reduce ongoing microvascular thrombosis in carefully selected patients without active bleeding [8].

The decision to administer therapeutic heparin in our patient was based on the presence of evolving purpuric lesions, laboratory evidence of DIC, markedly reduced protein C activity, and concern for progressive microvascular thrombosis despite preserved peripheral perfusion. Previous pediatric reports described variable outcomes with adjunctive heparin therapy. Hwang et al. reported two children with meningococcal PF treated with heparin in combination with antibiotics and blood product replacement; one survived with minimal sequelae, whereas the other died within 48 hours [15]. In our case, five days of therapeutic heparin were administered alongside aggressive support care without bleeding complications, and the patient survived without progression to tissue necrosis, vascular thrombosis, or limb ischemia. Although causality cannot be established, the favorable clinical course suggests that early anticoagulation may have contributed to limiting thrombotic progression in the setting of severe coagulopathy.

Previously reported survivors of meningococcal PF frequently develop significant long-term complications, including distal limb ischemia, digital amputations, orthopedic deformities, and chronic skin scarring [6]. Neurological complications, such as seizures and neurocognitive impairment, have also been described following severe meningococcal sepsis [16]. In contrast, our patient experienced a comparatively favorable outcome, with preserved limb perfusion, absence of gangrene or tissue necrosis, normal neuroimaging, and no neurological or orthopedic sequelae during follow-up. The early timing of presentation, prompt recognition of PF, rapid initiation of antimicrobial therapy, correction of coagulopathy, and multidisciplinary intensive care management likely mitigated progression to irreversible tissue injury.

This case highlights the importance of early recognition of meningococcal PF and demonstrates that timely multidisciplinary intervention may substantially alter disease trajectory even in the presence of severe coagulopathy and evolving DIC. It also supports the potential role of individualized anticoagulation therapy in carefully selected patients while emphasizing the need for continued long-term surveillance to identify delayed neurological and orthopedic complications.

## Conclusions

Meningococcal PF is a rare but highly aggressive pediatric emergency that requires rapid clinical recognition and immediate treatment. The presence of sepsis with rapidly progressive purpuric skin lesions and coagulopathy should prompt urgent antibiotic therapy, intensive supportive care, and correction of coagulation abnormalities. In this case, timely multidisciplinary management was associated with favorable outcomes, including survival without limb loss or major neurological sequelae. However, as a single case report, causality cannot be established, and favorable prognostic factors, such as presentation during compensated shock and relatively preserved platelet counts, may also have contributed to the outcome. Continued surveillance remains important to detect potential late complications, including growth plate disturbances. The role of therapeutic anticoagulation in meningococcal PF remains uncertain and warrants further study.
